# Male and Female Veterans’ Preferences for Eating Disorders Screening

**DOI:** 10.1007/s11606-022-07571-9

**Published:** 2022-08-30

**Authors:** Sabrina Hardin, Dawne Vogt, Brian N. Smith, Shannon Kehle-Forbes, Robin Masheb, Katherine M. Iverson, Karen Mitchell

**Affiliations:** 1 National Center for PTSD at VA Boston Healthcare System, Boston, MA USA; 2 Department of Psychiatry, Boston University School of Medicine, Boston, MA USA; 3 Minneapolis VA Healthcare System, Minneapolis, MN USA; 4 Department of Medicine, University of Minnesota, Minneapolis, MN USA; 5 VA Connecticut Healthcare System, West Haven, CT USA; 6 Department of Psychiatry, Yale School of Medicine, New Haven, CT USA

Eating disorders (EDs) are severe and costly mental illnesses that impact men and women of all ages. EDs are prevalent among veterans and affect as many as 9% of male veterans and 19% of female vetearns.^1^ Early detection can help mitigate high rates of morbidity and mortality associated with EDs;^2^ identifying factors that increase the likelihood that patient will disclose ED symptoms is essential. Individuals with EDs often experience elevated shame, which may affect willingness to disclose.^3^ Among patients who do not spontaneously disclose, many reveal eating problems when they are specifically queried by a healthcare professional.^2^ It is unknown whether this finding will extend to military veterans, as mental health screening within the Veterans Health Administration (VHA) has focused on service-related conditions. We compared preferences for ED screening between male and female veterans and veterans with and without probable EDs. We hypothesized that the majority of veterans would find ED screening acceptable and that provider knowledge and support would increase the likelihood of disclosure.

## METHODS

A national sample of 4126 randomly selected veterans were invited to complete an online survey; 1187 veterans completed the survey and are included in analyses. Women were oversampled to obtain a 1:1 ratio. A study fact sheet with elements of informed consent was included; survey completion indicated informed consent. Probable ED diagnoses were based on the Eating Disorders Diagnostic Scale for *DSM-5.*^4^ A modified scoring algorithm yielded a probable “any ED” variable (0 = no ED, 1 = probable ED); fasting and excessive exercise were excluded from the modified scoring algorithm due to a lack of reliability and validity data.^5^ To assess participants’ screening preferences, two sets of items were adapted from a study of intimate partner violence.^6^ Participants self-reported gender. Study procedures were approved by the local Institutional Review Board.

Statistics were computed using the “survey” and “svyVGAM” packages in R 4.0.0 with sample weights to account for non-response, enhance representativeness to the population, and obtain more accurate standard errors. We compared screening preferences between gender and ED diagnostic status groups, adjusting for age, using multinomial logistic regression models, with “unlikely” responses as the reference group.

## RESULTS

Participants were generally middle-aged (*M* = 53.9 years, *SD* = 13.79, range = 19–92), had a body mass index in the overweight range (*M* = 29.1, *SD* = 6.0), and were White (75.1%); 14.6% were Black, with smaller percentages of other racial groups, and 7.8% were Latinx. Participants identified as male (45.6%), female (50.0%), or other/missing (4.4%). In the full sample, 9.8% met criteria for a probable ED.

Table 1 includes results for the full sample and male and female subsamples. After adjusting for age, a higher proportion of men reported that they were likely to discuss their experiences with the provider, and to respond neutrally regarding whether they would be interested in hearing about resources that may be of help, compared to women.

**Table 1:** Screening Preferences in the Full Sample and by Gender

We just asked you a few questions about your eating habits and behaviors. If a healthcare provider, such as your primary care provider, were to ask you about such questions, to what extent would you be likely to^a^:															
	Unlikely (%) (reference group)			Neutral (%)						Likely (%)					
	Full	Women	Men	Full	Women	Men	*B*	OR	*p*	Full	Women	Men	*B*	OR	*p*
Be offended by such questions	82.0	73.6	83.4	12.4	16.9	12.0	0.33	1.39	0.15	5.6	9.5	4.6	0.66	1.93	0.06
Be comfortable answering these types of questions	21.5	23.8	21.5	15.6	17.6	14.6	−0.14	0.87	0.55	62.9	58.6	64.0	−0.37	0.69	0.05
Answer the questions honestly	7.7	7.9	7.6	6.6	8.7	59.8	0.14	1.15	0.72	85.7	83.4	86.4	−0.34	0.71	0.17
Appreciate that s/he is trying to help	7.1	6.9	7.2	16.5	17.2	16.4	−0.31	0.73	0.32	76.4	75.9	76.4	−0.21	0.81	0.43
Discuss your experiences with the provider	8.2	11.4	7.7	14.5	15.1	13.7	−0.53	0.59	0.08	77.3	73.5*	78.5*	−**0.57**	**0.57**	**0.01**
Be interested in hearing about resources that may be of help	13.1	14.4	12.8	25.4	20.0*	26.3*	−**0.55**	**0.58**	**0.03**	61.5	65.6	61.0	−0.04	0.96	0.85
If you engage in any of these eating behaviors (for example, overeating, night eating, making yourself vomit to counteract the effects of eating), how likely would you be willing to discuss your experience with your provider given the following factors^b^:															
	Unlikely (%) (reference group)			Neutral (%)						Likely (%)					
	Full	Women	Men	Full	Women	Men	*B*	OR	*p*	Full	Women	Men	*B*	OR	*p*
The provider is a woman	7.5	9.0	7.3	28.8	23.1	29.2	−0.47	0.63	0.14	63.7	67.9	63.5	−0.09	0.91	0.77
The provider explains that disordered eating is a common issue that impacts people’s health	6.3	7.3	6.2	31.5	27.5	31.8	−0.36	0.70	0.30	62.2	65.1	62.0	−0.06	0.94	0.86
The provider is supportive and nonjudgmental	5.3	6.1	5.4	26.2	21.1	26.9	−0.42	0.66	0.27	68.5	72.8	67.7	−0.05	0.95	0.75
The provider is knowledgeable about treatment and/or referral options	5.4	5.7	5.5	25.5	20.8	26.0	−0.52	0.59	0.20	69.1	73.5	68.5	−0.12	0.89	0.75
You were confident that your responses would be kept confidential	6.3	5.2	6.8	22.1	20.2	21.4	0.01	1.01	0.99	71.6	74.5	71.8	0.23	1.26	0.53
You were confident that your responses would not negatively impact your access to health care	7.2	5.9	7.7	24.5	20.2	24.4	−0.20	0.82	0.59	68.2	74.0	67.9	0.21	1.23	0.54

Note: ^a^Subsample sizes of participants from the full sample (*N* = 1187) who responded to each of the first set of screening preference item ranged from 1172 to 1174

^b^Subsample sizes of participants from the full sample (*N* = 1187) who responded to each of the second set of screening preference item ranged from 1087 to 1100

Multinomial logistic regression models with “unlikely” as the reference group were estimated to examine the association between gender (1 = male, 2 = female) and screening preferences, adjusting for age

*Significant (*p* < 0.05)

Table 2 presents results for screening preferences by ED status. After adjusting for age, participants with probable EDs were more likely to respond neutrally that they would be offended by ED screening questions or be comfortable answering questions compared to participants without EDs. Participants without EDs were more likely to report that they would answer the questions honestly and that confidentiality would increase their willingness to disclose, compared to participants with probable EDs. Participants without EDs also were more likely to respond neutrally to questions assessing whether confidentiality and whether the provider was a woman would increase willingness to disclose, relative to participants with probable EDs.

**Table 2:** Screening Preferences by Eating Disorder Case Status

We just asked you a few questions about your eating habits and behaviors. If a healthcare provider, such as your primary care provider, were to ask you about such questions, to what extent would you be likely to^a^:												
	Unlikely % (reference group)		Neutral %					Likely %				
	ED	Non-ED	ED	Non-ED	*B*	*OR*	*p*	ED	Non-ED	*B*	*OR*	*p*
Be offended by such questions	51.0	85.4	26.4*	10.9*	**1.33**	**3.78**	**0.001**	22.6*	3.7*	**2.26**	**9.58**	**< 0.001**
Be comfortable answering these types of questions	18.7	21.8	35.5*	13.4*	**1.01**	**2.75**	**<0.01**	45.8	64.8	−0.35	0.70	0.32
Answer the questions honestly	12.3	7.2	13.7	5.8	0.15	1.16	0.79	74.0*	87.0*	−**0.91**	**0.40**	**0.02**
Appreciate that s/he is trying to help	10.6	6.7	20.2	16.1	−0.48	0.62	0.38	69.2	77.2	−0.76	0.47	0.08
Discuss your experiences with the provider	10.9	7.9	19.9	13.9	−0.14	0.87	0.79	69.2	78.1	−0.44	0.64	0.25
Be interested in hearing about resources that may be of help	12.1	13.2	34.6	24.4	0.38	0.68	0.42	53.3	62.4	0.03	1.03	0.93
If you engage in any of these eating behaviors (for example, overeating, night eating, making yourself vomit to counteract the effects of eating), how likely would you be willing to discuss your experience with your provider given the following factors^b^:												
	Unlikely % (reference group)		Neutral %					Likely %				
	ED	Non-ED	ED	Non-ED	*B*	*OR*	*p*	ED	Non-ED	*B*	*OR*	*p*
The provider is a woman	15.3	6.5	22.8*	29.6*	−**1.06**	**0.35**	**0.03**	61.9	63.9	−0.79	0.45	0.07
The provider explains that disordered eating is a common issue that impacts people’s health	13.5	5.4	25.2	32.2	−1.07	0.34	0.05	61.3	62.4	−0.83	0.44	0.07
The provider is supportive and nonjudgmental	9.6	4.8	19.9	26.9	−1.09	0.34	0.06	70.6	68.3	−0.71	0.49	0.15
The provider is knowledgeable about treatment and/or referral options	7.3	5.2	22.6	25.9	−0.77	0.46	0.22	70.2	69.0	−0.55	0.58	0.29
You were confident that your responses would be kept confidential	14.6	5.4	16.0*	22.8*	−**1.67**	**0.19**	**0.01**	69.4*	71.8*	−**1.24**	**0.29**	**0.02**
You were confident that your responses would not negatively impact your access to health care	12.2	6.6	20.9	25.0	−1.13	0.32	0.06	66.9	68.4	−0.89	0.41	0.06

Note: ^a^Subsample sizes of participants from the full sample (*N* = 1187) who responded to each of the first set of screening preference item ranged from 1172 to 1174

^b^Subsample sizes of participants from the full sample (*N* = 1187) who responded to each of the second set of screening preference item ranged from 1087 to 1100

The subsample sizes of participants meeting probable criteria for an ED were 106 women and 40 men

*ED* eating disorder. Participants were coded as having “any ED” if they met probable criteria for anorexia nervosa, bulimia nervosa, binge eating disorder, atypical anorexia nervosa, subthreshold bulimia nervosa or binge eating disorder, purging disorder, or night eating syndrome according to the Eating Disorder Diagnostic Scale-5

Multinomial logistic regression models, with “unlikely” as the reference group were estimated to examine the association between ED status (1 = ED, 2=no ED) and screening preferences, adjusting for age

*Significant (*p* < 0.05)

## DISCUSSION

The finding that most veterans are amenable to screening for EDs should be reassuring to clinicians and emphasizes the relevance of ED screening to VHA. There were few gender differences in screening preferences or differences between participants with and without probable EDs. Findings further indicate that to optimize the utility of ED screening, clinicians should emphasize confidentiality, be knowledgeable about treatment and referral options, and be non-judgmental. A strength of the study is the nearly equal representation of male and female veterans. Limitations include use of a self-report measure to determine probable ED diagnoses, lack of validation of the screening preferences measure for EDs, and the predominantly White sample. Examining the effectiveness of VHA ED screening and referral in the context of expanding access to ED specialty treatment is warranted.
